# Complications Post-COVID-19 and Risk Factors among Patients after Six Months of a SARS-CoV-2 Infection: A Population-Based Prospective Cohort Study

**DOI:** 10.3390/epidemiologia3010006

**Published:** 2022-02-10

**Authors:** Salvador Domènech-Montoliu, Joan Puig-Barberà, Maria Rosario Pac-Sa, Paula Vidal-Utrillas, Marta Latorre-Poveda, Alba Del Rio-González, Sara Ferrando-Rubert, Gema Ferrer-Abad, Manuel Sánchez-Urbano, Laura Aparisi-Esteve, Gema Badenes-Marques, Belen Cervera-Ferrer, Ursula Clerig-Arnau, Claudia Dols-Bernad, Maria Fontal-Carcel, Lorna Gomez-Lanas, David Jovani-Sales, Maria Carmen León-Domingo, Maria Dolores Llopico-Vilanova, Mercedes Moros-Blasco, Cristina Notari-Rodríguez, Raquel Ruíz-Puig, Sonia Valls-López, Alberto Arnedo-Pena

**Affiliations:** 1 Emergency Service Hospital de la Plana, 12540 Villarreal, Castellon, Spain; pttcarmen@hotmail.com (S.D.-M.); martalapo@hotmail.com (M.L.-P.); manu.msu@gmail.com (M.S.-U.); gemabamar@hotmail.com (G.B.-M.); belencerveraferrer@hotmail.es (B.C.-F.); ursuclerig@gmail.com (U.C.-A.); lornagl78@gmail.com (L.G.-L.); jovasal1987@gamil.com (D.J.-S.); llopivila@hotmail.com (M.D.L.-V.); notari_cri@gva.es (C.N.-R.); raquelruizpuig@gmail.com (R.R.-P.); Sonia.valls.lopez@gmail.com (S.V.-L.); 2 Vaccines Research Area FISABIO, 46020 Valencia, Spain; jpuigb55@gmail.com; 3 Public Health Center, 12003 Castelló de la Plana, Castellon, Spain; charopac@gmail.com; 4 Health Centers I and II, 12530 Borriana, Castellon, Spain; vidalutrillaspaula@gmail.com (P.V.-U.); delrio_alb@gva.es (A.D.R.-G.); sfr1812@gmail.com (S.F.-R.); gferrer@uji.es (G.F.-A.); mercedesmb1094@gmail.com (M.M.-B.); 5 Carinyena Health Center, 12540 Villarreal, Castellon, Spain; lauraaparisiesteve@gmail.com; 6 Health Center, 12200 Onda, Castellon, Spain; claudiadb1294@hotmail.com; 7 Health Center, 12600 La Vall d’Uixó, Castellon, Spain; fontalcarcel93@hotmail.com; 8 Villa Fátima School, 12530 Borriana, Castellon, Spain; carmendole@hotmail.com; 9 Department of Health Science, Public University Navarra, 31006 Pamplona, Navarra, Spain; 10 Epidemiology and Public Health (CIBERESP), 28029 Madrid, Spain

**Keywords:** COVID-19, incidence, complications, symptoms, recovery, health, risk factors, exposure, cohort, prospective, population-based

## Abstract

In October 2020, we conducted a population-based prospective cohort study to determine post-COVID-19 complications, recovery, return to usual health, and associated risk factors in 536 cases of COVID-19 outbreak in Borriana (Spain) by administering an epidemiological questionnaire via phone interviews. A total of 484 patients participated (90.3%), age mean 37.2 ± 17.1 years, and 301 females (62.2%). Mild illness was the most common COVID-19 manifestation. After six months, 160 patients (33.1%) suffered at least one complication post-COVID-19, and 47 (29.4%) of them sought medical assistance. The most frequent persistent symptoms were hair loss, fatigue, loss of smell or taste, and headache. Risk factors associated with a complication were female sex (adjusted relative risk, [aRR] = 1.93 95% confidence interval [CI] 1.41–2.65), age 35 years and above (aRR = 1.50 95% CI 1.14–1.99), B blood group (aRR = 1.51 95% CI 1.04–2.16), current smoker (RR = 1.61 95% CI 1.02–2.54), and at least a COVID-19 exposure (aRR = 2.13 95% CI 1.11–4.09). Male sex, age younger than 35 years, and low COVID-19 exposures were associated with better recovery and return to usual health. A third of patients presented persistent symptoms compatible with the long-COVID-19 syndrome. In conclusion, an active medical follow-up of post-COVID-19 patients must be implemented.

## 1. Introduction

After more than year of the COVID-19 pandemic, many aspects of this new and complicated disease are still poorly understood and characterized [1,2], such as the frequency and risk factors associated with complications or sequelae after acute COVID-19 illness. Additionally, it is not clear what should be considered a long-COVID-19 or post-COVID-19 syndrome (LCS) [3,4,5]. Currently, post-COVID-19 syndrome is defined by persistent clinical signs and symptoms that appear while or after suffering COVID-19 for more than 12 weeks and that cannot be explained by an alternative diagnosis [6,7,8,9]. The syndrome includes affectation of respiratory, cardiovascular, neurological, gastrointestinal, musculoskeletal systems, psychiatric/psychological issues, ear, nose, throat, and dermatological symptoms, and general status impairment [4,5,6,7,10,11,12]. However, it is controversial whether LCS should be considered a post-infectious syndrome rather than a singular syndrome in the evolution of the disease [13]. To avoid controversies, recent research focuses on LCS consensus definition [14], and underlines the prolonged effects of post-acute COVID-19 symptomatology and organ dysfunction [15,16], included a post-infectious myalgic encephalomyelitis/chronic fatigue syndrome which could happen after viral diseases [17,18].

The frequency of complications subsequent to the acute phase has important variations in the ranges from 10% to 93% [3,5,8,19,20,21,22,23,24,25]. The studies of post-COVID-19 complications are heterogeneous; including hospitalized versus non-hospitalized patients, with variable demographic characteristics of patients, the severity of the disease, follow-up time, different measures of the complications, control groups for comparison, designs, sample size, and statistical analysis [5,21,26,27,28,29]. In this situation, accruing evidence on the frequency and the manifestation of LCS and related risk factors is needed [22,30,31].

Our aim was to describe post-COVID-19 complications, recovery, and return to the usual state of health in a cohort of patients that suffered a SARS-CoV-2 infection and estimate the strength of the association of diverse risk factors with LCS.

## 2. Materials and Methods

### 2.1. Explanation

In March 2020 a COVID-19 outbreak took place during the mass gathering events (MGEs) of the *Falles* festival in Borriana, a city of 34,000 inhabitants, in the Castellon province, Valencia Community (Spain), with 536 laboratory-confirmed cases of COVID-19 [32]. In October 2020 a population-based prospective cohort study of 536 COVID-19 patients was performed by the Public Health Center of Castelló de la Plana, and the Emergency and Microbiology and Clinical Analysis Services of Hospital de la Plana Vila-real [33].

We first performed a sero-survey and an epidemiological questionnaire survey in June 2020 that has been described by Domenech-Montoliu and co-authors [32]. A second sero-survey and questionnaire were run in October 2020. In summary, the sero-surveys included determinations of anti-SARS-CoV-2 N-antibodies in June and October 2020 with a flow chart of the study, and ABO blood groups and vitamin D status in October 2020. The sero-survey results were subject of two previous publications [33,34], and are no further discussed in the current manuscript. Health staff of Hospital La Plana Vila-real, and several health centers (Borriana, Vila-real, Onda, and Vall D’Uixo) performed the telephone interviews. In June 2020, we collected information about acute COVID-19 illness (symptoms, medical consultation, health status), demographic data (age, sex), lifestyle (smoking habit, alcohol consumption, physical exercise), body mass index (BMI = kg/m^2^, obesity ≥ 30.0), occupation as a proxy social class, COVID-19 exposure (measured as positive observation a person coughing at the MGEs, contact with a COVID-19 patient or a relative with COVID-19, attendance two or more MGEs). In the second (October 2020) telephone survey, we collected information on the evolution of the COVID-19 disease, complications, persistent symptoms, health recovery, return to the usual state of health, and medical consultation after the acute phase.

### 2.2. Statistical Methods

We performed the statistical analysis and considered complications, recovery, and return to the usual state of health as dependent variables; age, sex, ABO blood groups, occupation, chronic disease, lifestyle, BMI, COVID-19 exposure during the MGEs, symptoms, and medical consultation of acute COVID-19 illness were independent variables.

In the univariate analysis, Chi2 and Fisher exact tests were used to compare qualitative variables and the Kruskal-Wallis test for quantitative variables. The incidence rate (IR) of a variable was obtained dividing the positive cases of this variable by the exposed population, and the relative risk (RR) of each independent variable, dividing the IR of the population exposed by the IR of the population no-exposed of this variable, with a 95% confidence interval (CI). After a revision of medical literature, we employed directed acyclic graphs (DAGs) [35], and the program DAGitty 3.0 version (Johannes Textor, Nijmegen, The Netherlands) [36,37] to define the potential confounders of each independent variable. We used inverse probability weighted regression [38] in the multivariable analysis to estimate adjusted incidence rates (aIR) and adjusted relative risks (aRR). We applied Stata^®^ 14.2 version (Stata Corp, College Station, TX, USA) or all calculations.

The study was part of public health surveillance activities of the COVID-19 outbreak control measures in the MGEs of the *Falles* festival [32], and it was exempt from the Ethics Review Board approval’s protocol according to the Spanish legislation and regulations [39,40,41,42]. The study was approved by the director of the Public Health Center of Castelló de la Plana and the management of the Health Department of la Plana. All participants or the parents of minors provided the informed written consent to be included in the study.

## 3. Results

A total of 484 patients participated in the study with a response rate of 90.3% from the 536 COVID-19 laboratory-confirmed cases in MEGs. The mean ± standard deviation (SD) age of participants was 37.2 ± 17.1 (range 1–81) years, and 301 were females (62.2%). In the outbreak, a death attributable to COVID-19 was reported, 13 patients required hospitalization, and mild illness was the most frequent clinical presentation.

Patients’ characteristics and potential risk factors of complications of the COVID-19 illness, health recovery, and return to the usual health before the acute illness in the six months of follow-up are shown in Table 1. A total of 160 participants reported at least one post-COVID-19 complication (33.1%), 47 (29.4%) of them sought medical care, but no hospitalizations took place. An 81.8% of the participants recovered, and 83.2% returned to their usual state of health before the COVID-19 episode. Complications were more frequent in females, elder age, B blood group, current smoking status, high COVID-19 exposure, and those who reported symptomatic acute COVID-19 episode, and sought medical care. Health recovery and return to the usual health were highest in males, young individuals, attendance of MEGs less than two events, asymptomatic acute illness, and fewer medical consultations.

We show persistent post-COVID-19 symptoms by sex and four age groups in Table 2. The most frequent signs or symptoms were hair loss (24.8%), fatigue (17.4%) loss of smell or taste (16.1%), headache (15.1%) muscle pain (11.8%), insomnia (11.8%), anxiety (9.1%), weakness (8.5%), restlessness (8.1%), hands and foot’s pain (7.4), and dyspnea (6.8%). Females reported suffering more symptoms than males, especially hair loss (35.9% versus 6.5% *p* = 0.000), fatigue (21.9% versus 9.8% *p* = 0.000), and loss of smell or taste (21.6% versus 7.1% *p* = 0.000).

The mean (±SD) duration of persistent post-COVID-19 symptoms was 160.9 ± 45.5 days (range of 3–280 days). The mean (±SD) duration in females was 162.6 ± 44.5 (range 3–280) days, and in males 155.8 ± 48.8 (range 15–210) days without significant difference (*p* = 0.444).

Patients 65 years old and older reported more symptoms, with the exception of loss of smell or taste and the hair loss, which were more common in subjects in the range of 15–34 years and 35–64 years. In subjects 65 years and older, the most frequently reported symptoms were fatigue (41.2%), hand/foot pain (29.4%), and insomnia (29.4%).

The mean duration of post-COVID-19 symptoms was 157.5 ± 45.0 days, 163.3 ± 53.9 days, 160.6 ± 42.3 days, and 154.3 ± 43.9 days for subjects aged 0–14, 15–34, 35–64, and 65 and above, with *p* > 0.05 for the difference.

Considering the aIR of acute COVID-19 symptoms and the occurrence of complications (Table 3), many of acute symptoms were predictive of complications, highlighting: weakness (aRR = 2.25 95% CI 1.62–3.13), fever (aRR = 1.79 95% CI 1.35–2.38), loss of smell or taste (aRR = 1.47 95% CI 1.11–1.94), headache (aRR = 1.53 95% CI 0.78–1.99), myalgia (aRR = 1.50 95% CI 1.13–1.99), dyspnea (aRR = 1.61 95% CI 1.00–2.59), and skin’s lesions (aRR = 1.84 95% CI 1.41–2.40). Symptomatic patients presented an elevated risk of complications versus asymptomatic patients (aRR = 4.60 95% CI 2.05–10.3), and 5 acute symptoms and above were associated with a high risk of complications (aRR = 1.80 95% CI 1.37–2.36). Medical consultation increased the risk of complications (aRR = 2.61 95% CI 1.95–3.50).

Symptoms of acute illness and health recovery are shown in Table 4. Not to report dyspnea was associated with recovery (aRR = 1.81 95% CI 1.10–2.99). The absence of loss of smell or taste (aRR = 1.13 95% CI 1.03–1.24), weakness (aRR = 1.12 95% CI 1.02–1.22), headache (aRR = 1.10 95% CI 1.01–1.21), and less than five acute symptoms were associated with recovery (aRR = 1.15 95% CI 1.04–1.27). Not to report medical consultations was associated with recovery (aRR = 1.28 95% CI 1.15–1.42).

The absence of weakness during the acute illness was associated with return to the usual health (aRR = 1.15 95% CI 1.6–1.20) Table 5. The absence of loss of smell or taste (aRR = 1.10 95% CI 1.01–1.1), headache (aRR = 1.10 95% CI 1.01–1.20), less than 5 acute illness symptoms were associated with increased return to the usual health (aRR = 1.13 95% CI 1.03–1.24), as was the absence of medical consultation (aRR = 1.22 95% CI 1.12–1.35).

In the adjusted multivariable analysis (Table 6), risk factors associated with a complication were female sex (aRR = 1.93 95% CI 1.41–2.65), age-group 35 years and above (aRR = 1.50 95% CI 1.14–1.99), B blood group versus O blood group (aRR = 1.51 95% CI 1.04–2.16), and current smokers versus ex-smokers (aRR = 1.61 95% CI 1.02–2.54). Three of five measures of COVID-19 exposure presented a high risk of complications: observe a person with a cough at MGEs (aRR = 1.38 95% CI 1.05–1.81), attendance MGEs ≥ 2 (aRR = 1.42 95% CI 1.04–1.94), and at least a COVID-19 exposure (aRR = 2.13 95% CI 1.11–4.09).

Considering health recovery after the acute illness (Table 7), younger age than 35 years (aRR = 1.15 95% CI 1.06–1.25), and male sex (aRR = 1.09 95% CI 1.00–1.19) were associated with more recovery (Table 7). The AB blood group subjects reported 100% recovery. Attendance with less than two MGEs was associated with more recovery (aRR = 1.11 95% CI 1.02–1.21).

The return to the usual health was associated with male sex (aRR = 1.11 95% CI 1.02–1.20), and age 0–34 years (aRR = 1.14 95% CI 1.05–1.24), no contact with a COVID-19 patient (aRR = 1.15 95% CI 1.06–1.25), and AB blood group (aRR = 1.15 95% CI 1.09–1.21) (Table 8).

## 4. Discussion

Our results suggest that a third of COVID-19 patients with mild illness experienced post-COVID-19 symptoms six months after the acute episode. The most frequent symptoms were loss of hair, fatigue, and loss of smell or taste. Acute symptoms of illness, female sex and elder age were associated with complications, and a less frequent recovery and return to usual health. As highlighted findings of our study, several exposures to COVID-19, including contact with a COVID-19 patient, attendance to two or more MGEs, and observing a person with a cough at MEGs, were risk factors of complications. On the other hand, lower COVID-19 exposures were associated with better recovery and return to the usual health. These results are in line with other studies that found COVID-19 exposures were associated with post-traumatic stress symptoms of COVID-19 patients, compatible with LCS [43,44,45].

Complications of COVID-19 have been observed in many prospective cohort studies indicating the importance of a follow-up of COVID-19 patients [19,46,47,48]. In general, the frequency and severity of these complications or “sequelae” are associated with acute COVID-19 illness [4,31,49], and our results are consistent with these studies, these being hair loss, fatigue, and loss of smell or taste such as more frequent reported persistent symptoms in a multi-system affectation [21,46,50,51,52,53]. Considering that our cohort had few hospitalized patients, the incidence of complications was lower in comparison with other cohort studies [49,51,52]. Recovery and return to the usual health were higher than those found by studies in hospitalized and non-hospitalized patients, and medical consultation was lower [54,55]. Soraas and co-authors 2021 [56] analyzed a cohort of non-hospitalized COVID-19 patients, and found 36% reported worse health status one year after illness. Persistent symptoms in cohorts of non-hospitalized patients presented large variations in this percentage, ranging from 20% to 61%, possible due to differences in the duration of follow-up, age and sex distributions, and sample sizes [57,58].

Several symptoms during acute illness were predictive of the post-COVID-19 persistence of symptoms, including weakness, skin lesions, loss of smell or taste, headache, and more of five acute symptoms, in line with cohort studies of non-hospitalized patients [31,51,56,59], but not others [60]. In a study in Norway, fatigue was present in 46% of non-hospitalized COVID-19 patients after a mean of four months; persistence of fatigue was associated with female sex, high numbers of symptoms in the acute illness, and confusion [61]. In addition, dyspnea increased the possibility of post-COVID-19 persistent symptoms and poor health recovery.

We found that females and elder patients experienced a higher frequency of post-COVID-19 symptoms compared with males and young individuals, as has been found in several studies [19,22,31,49,51,61]. The highest incidence of symptoms in females is not well understood and could be associated with the immune viral response [53,56]. Older age can worsen the outcome considering the inflammatory and immunodeficiency state of aging [61,62,63]. This poor recovery could also be associated with a more frequent severe course of the disease, probably more COVID-19 exposures, and a post-viral disease syndrome which could increase with age [64].

Another associated factor with persistent symptoms was the B blood group compared to the O blood group. A more severe course of COVID-19 disease in the B-group has been described including thrombotic complications [65,66,67]. The causes of the B-group having more complications compared to the O-group are unknown. Some studies have found that the O-group presents more resistance to some infectious diseases [68], higher physiologic capacity [69], low levels of SARS-CoV-2 IgG compared to non-O groups [70], and some protection from the anti-A/anti-B presence and furin cleavage [71]. In our study [34] the persistence of SARS-CoV-2 antibodies was lower in the O-group, and it has been found that B-group presented higher SARS-CoV-2 neutralizing antibody titers than the other ABO [72].

Current smoking status was associated with more complications, but it was not associated with heath recovery and return to the usual health. In some studies, current smoking is a risk factor of illness severity, but there are different results with respect to complications [56,59,73,74,75,76]. However, the incidence of COVID-19 in current smokers is lower than in non-smokers [77].

We have not observed an association between BMI, obesity, regular exercise, or alcohol consumption and post-COVID-19 symptoms, in contrast with other studies [31,53]. In addition, high levels of anti-SARS-CoV-2 antibodies or their increase between June 2020 and October 2020 were not associated with complications, recovery, and return to the usual health [33], contrary to other studies [25,28,57]. However, attendance of MGEs, observing a person with a cough at MGEs, and contact with a COVID-19 patient were associated with complications, inferior recovery, and less frequent return to usual health. All of the above highlights the importance of intensity of COVID-19 exposure, duration, viral load, and the place where the exposure occurred [78,79,80,81]. In experimental models, the differences between the persistence of virus infection and the quantity of inoculum have been found [82].

Our study has some strengths such as its prospective design, a population-based approach, a high participation rate, a method to explore potential confounding variables [37], and the use of multivariate analysis in order to estimate adjusted risk factors for different variables. However, these results could be more accurate. In addition, we studied not only complications, but also health recovery and return to a usual state of health.

The study limitations include the use of a questionnaire to ascertain reported complications and symptoms of post-COVID-19 disease, with no medical examination of patients. As a consequence, we cannot exclude information bias even if the questionnaires were administrated by physicians and nurses. Most of the patients suffered a mild COVID-19 illness, and the participant population is not representative of severe COVID-19 disease. We cannot discard a residual confounding despite the analysis. COVID-19 is a new disease and some unknown variables not-collected in our study could have an impact on our observations.

The causes of LCS are unknown, but some aspects could be considered, including viral persistence, post-infectious myalgic encephalomyelitis/chronic fatigue syndrome, metabolism alteration, disproportionate autoimmunity, pathological inflammation, disruption of the autonomic nervous system, post-traumatic stress, underlying chronic disease, damage to the lungs, brain, heart, kidney, and other organs [5,17,18,26,83,84,85,86].

Considering the high incidence of persistence of COVID-19 related symptomatology found after COVID-19 infections, some points should be addressed in order to improve the prevention and management of the LCS, such as the medical follow-up of LCS patients to establish their natural history, determine laboratory tests needed, validate functional scales, accrue more information of potential risk factors, and apply appropriate therapies [22,59,84,87,88]. Our prospective design and the population-based approach have advantages in obtaining results with less bias than other designs.

## 5. Conclusions

Despite a majority of mild illness, a third of patients presented persistent symptoms compatible with the LCS, and several risk factors were found. An active medical follow-up of post-COVID-19 patients must be implemented.

## Figures and Tables

**Table 1:** Incidence rate (IR) of complications, health recovery, and return to the usual health. Borriana COVID-19 cohort 2020.

Variables	Total	Complications	Health Recovery	Return to the Usual Health
	N (%)	N IR ^1^ (%)	N IR ^1^ (%)	N IR ^1^ (%)
Population	484	160 (33.1)	395(81.8) ^2^	402 (83.2) ^3^
Sex				
Female	301 (62.1)	119 (39.5)	240 (79.7)	243 (80.7)
Male	183 (37.8)	41 (22.4)	155 (85.2)	159 (87.4)
Age-groups (years)				
0–14	53 (11.0)	5 (9.4)	51 (96.2)	51 (96.2)
15–34	149 (30.8)	51 (34.2)	127 (85.2)	129 (86.6)
34–64	264 (54.5)	97 (36.6)	206 (77.7)	211 (79.6)
65 and above	18 (3.7)	7 (41.2)	11 (68.8)	11 (68.8)
ABO blood group ^4^				
O	200 (41.1)	63 (31.5)	166 (83.0)	173 (86.5)
A	220 (45.6)	70 (31.8)	179 (81.4)	178 (80.9)
B	44 (9.1)	22 (50.0)	31 (72.1)	32 (74.4)
AB	19 (3.9)	4 (21.1)	19 (100.0)	19 (100.0)
Occupations ^5,6^				
I–II	145 (30.2)	48 (33.1)	123 (84.8)	125 (86.2)
III–VI	336 (69.9)	112 (33.3)	269 (80.3)	274 (81.8)
Chronic disease ^7^	166 (40.1)	63 (38.0)	123 (74.6)	127 (77.0)
Smoking ^8^				
No-smoking	297 (63.5)	98 (33.0)	249 (84.1)	252 (85.1)
Ex-smoking	106 (22.7)	36 (34.0)	80 (75.5)	80 (75.5)
Current smoking	65 (13.9)	23 (35.4)	51 (78.5)	56 (86.2)
Alcohol consumption ^9^	108 (23.0)	34 (31.5)	85 (78.7)	88 (81.5)
Physical exercise	289 (59.7)	95 (32.9)	236 (81.9)	243 (84.4)
Body Mass Index (BMI) (kg/m^2^) ^10^				
<18.5	41 (8.6)	6 (14.6)	40 (97.7)	41 (100.0)
18.5–24.9	210 (43.8)	82 (39.1)	165 (78.6)	171 (81.4)
25.0–29.9	148 (30.9)	48 (32.4)	123 (83.7)	120 (81.6)
≥30.0	80 (16.7)	23 (28.8)	63 (78.7)	66 (82.5)
Observed a person with a cough at MGEs ^11,12^	203 (42.5)	78 (38.0)	166 (81.0)	165 (80.5)
Contact with COVID-19 patient ^13^	390 (81.8)	138 (35.4)	314 (80.7)	316 (81.2)
Family with COVID-19 patient ^14^	303 (62.7)	110 (36.3)	243 (80.5)	246 (81.5)
Attendance MGEs ≥ 2	295 (61.0)	115 (39.0)	228 (77.6)	235 (79.9)
At least a COVID-19 exposure ^15^	455 (94.0)	155 (34.1)	370 (81.5)	375 (82.6)
Symptomatic patients of COVID-19 illness	430 (88.8)	155 (34.1)	344 (80.0)	350 (81.4)
Asymptomatic patients	54 (11.2)	5 (9.3)	51 (94.4)	52 (96.3)
Medical consultation of acute COVID-19 illness				
Yes	208 (43.0)	106 (51.0)	147 (71.0)	150 (72.5)
Medical care post-COVID-19 periode ^16^				
Yes	47 (9.8)	47 (100.0)	19 (40.4)	20 (42.6)

^1^ IR, Incidence rate; ^2^ Missing information 1 participant; ^3^ Missing information 1 participant; ^4^ Missing information 1 participant; ^5^ Occupation groups I–II professional, managerial, and technical occupations; groups III–VI: skilled, no-manual or manual, partly skilled, unskilled occupations; ^6^ Missing information 3 participants; ^7^ Missing information 4 participants; ^8^ Missing information 16 participants; ^9^ Missing information 14 participants; ^10^ Missing information 5 participants; ^11^ MGEs, mass gathering events; ^12^ Missing information 6 participants; ^13^ Missing information 7 participants; ^14^ Missing information 1 participant; ^15^ Summary all exposures; ^16^ Missing information 5 participants.

**Table 2:** Reported persistent post-COVID-19 symptoms of participants by sex and age. Borriana COVID-19 cohort 2020.

Persistent Symptoms	Male N = 183	Female N = 301	Total N = 484	*p*-Value	Age-Groups (Years)				*p*-Value
					0–14	15–34	35–64	≥65	
	N (%)	N (%)	N (%)		N (%)	N (%)	N (%)	N (%)	
Fatigue	18 (9.8)	66 (21.9)	84 (17.4)	0.007	1 (1.9)	25 (16.8)	55 (20.8)	7 (41.2)	0.000
Weakness	11 (6.0)	30 (10.0)	41 (8.5)	0.177	0 (0)	10 (6.7)	28 (10.6)	3 (17.7)	0.012
Dyspnea	12 (6.6)	21 (7.0)	33 (6.8)	1.000	0 (0)	4 (4.0)	24 (9.1)	3 (17.7)	0.006
Thorax oppression	5 (2.7)	13 (4.3)	18 (3.7)	0.463	0 (0)	5 (3.4)	9 (3.4)	4 (23.5)	0.005
Cough	10 (5.5)	17 (5.7)	27 (5.6)	1.000	1 (1.9)	7 (4.7)	16 (6.0)	3 (17.7)	0.118
Fever	0 (0)	5 (1.7)	5 (1.0)	0.162	1 (1.9)	2 (1.3)	2 (0.8)	0 (0)	0.594
Throat pain	5 (2.7)	23 (7.6)	28 (5.8)	0.027	3 (5.7)	9 (6.0)	15 (5.7)	1 (5.9)	1.000
Runny nose	15 (8.2)	22 (7.3)	37 (7.6)	0.727	5 (9.4)	10 (6.7)	21 (7.9)	1 (5.9)	0.905
Loss of smell/taste	13 (7.1)	65 (21.6)	78 (16.1)	0.000	1 (1.9)	25 (16.8)	49 (18.5)	3 (17.7)	0.007
Nausea/vomits	3 (1.6)	4 (1.3)	7 (1.4)	1.000	1 (1.9)	1 (0.7)	5 (1.9)	0 (0)	0.637
Diarrhea	11 (6.0)	10 (3.3)	21 (4.3)	0.173	2 (3.8)	8 (5.4)	11 (4.7)	0 (0)	0.952
Alimentary intolerance	2 (1.1)	8 (2.7)	10 (2.1)	0.332	0 (0)	4 (2.7)	6 (2.3)	0 (0)	0.758
Abdominal pain	6 (3.3)	14 (4.7)	20 (4.1)	0.839	2 (3.8)	4 (2.7)	13 (4.9)	1 (5.9)	0.581
Muscle pain	13 (7.1)	44 (14.6)	57 (11.8)	0.013	0 (0)	9 (6.0)	44 (16.6)	4 (23.5)	0.000
Headache	18 (9.8)	55 (18.3)	73 (15.1)	0.013	2 (3.8)	25 (16.8)	44 (16.6)	2 (11.8)	0.065
Hands/foots pain	5 (2.7)	31 (10.3)	36 (7.4)	0.002	1 (1.9)	4 (2.7)	26 (9.8)	5 (29.4)	0.000
Dizziness	6 (3.3)	16 (5.3)	22 (4.5)	0.371	0 (0)	9 (6.0)	12 (4.5)	1 (5.9)	0.255
Ringing ears	8 (4.4)	15 (5.0)	23 (4.8)	0.829	0 (0)	4 (2.7)	16 (6.0)	3 (17.7)	0.014
Disorder vision	5 (2.7)	13 (4.3)	18 (3.7)	0.463	0 (0)	3 (2.0)	15 (5.7)	0 (0)	0.111
Insomnia	15 (8.2)	42 (14.0)	57 (11.8)	0.060	2 (3.8)	14 (9.4)	36 (13.6)	5 (29.4)	0.019
Night sweats	5 (2.7)	24 (8.0)	29 (6.0)	0.018	1 (1.9)	6 (4.0)	18 (6.8)	4 (23.5)	0.019
Depression	3 (1.6)	12 (4.0)	15 (3.1)	0.183	1 (1.9)	1 (0.7)	11 (4.2)	2 (11.8)	0.034
Restlessness	7 (3.8)	32 (10.6)	39 (8.1)	0.009	0 (0)	9 (6.0)	27 (10.2)	3 (17.7)	0.011
Difficulty concentration	4 (2.2)	16 (5.3)	20 (4.1)	0.104	0 (0)	5 (3.4)	13 (4.9)	2 (11.8)	0.114
Anxiety	9 (4.9)	35 (11.6)	44 (9.1)	0.014	0 (0)	13 (8.7)	28 (10.6)	3 (17.7)	0.019
Mental confusion	2 (1.1)	14 (4.7)	16 (3.3)	0.037	0 (0)	4 (2.7)	10 (3.8)	2 (11.8)	0.124
Difficulty articulating words	1 (0.6)	5 (1.7)	6 (1.2)	0.416	0 (0)	1 (0.7)	4 (1.5)	1 (5.9)	0.261
Difficulty to solve math operations	1 (0.6)	3 (1.0)	4 (0.8)	1.000	0 (0)	0 (0)	4 (1.5)	0 (0)	0.505
Skin’s lesions	9 (4.9)	16 (5.3)	25 (5.2)	1.000	2 (3.8)	5 (3.4)	17 (6.4)	1 (5.9)	0.518
Loss hair	12 (6.5)	108 (36.0)	120 (24.8)	0.000	3 (5.7)	48 (32.2)	64 (24.2)	5 (29.4)	0.001

**Table 3:** Symptoms of acute COVID-19 illness and complications by adjusted incidence rate (aIR) and adjusted relative risk (aRR). Borriana COVID-19 cohort 2020.

Symptoms	Complications		
	aIR ^1^ (%)	aRR (95% CI)	*p*-Value
Cough			
Yes	38.2	1.27 (0.98–1.63)	0.067
No	30.2	1.00	
Runny nose Yes	34.3	1.05 (0.79–1.38)	0.756
No	32.8	1.00	
Throat pain Yes	37.5	1.17 (0.89–1.52)	0.255
No	32.1	1.00	
Fever Yes	40.7	1.79 (1.35–2.38)	0.000
No	22.7	1.00	
Loss of smell/taste Yes	39.0	1.47 (1.11–1.94)	0.006
No	26.6	1.00	
Diarrhea Yes	38.5	1.26 (0.96–1.67)	0.094
No	30.5	1.00	
Vomits Yes	44.7	1.38 (0.91–2.08)	0.128
No	32.5	1.00	
Weakness Yes	41.1	2.25 (1.62–3.13)	0.000
No	8.3	1.00	
Headache Yes	40.7	1.53 (1.78–1.99)	0.002
No	26.6	1.00	
Myalgia Yes	39.7	1.50 (1.13–1.99)	0.005
No	26.4	1.00	
Dyspnea Yes	61.2	1.61 (1.00–2.59)	0.048
No	47.7	1.00	
Skin’s lesions Yes	58.0	1.84 (1.41–2.40)	0.000
No	31.5	1.00	
Number of symptoms ≥ 5 Yes	43.8	1.80 (1.37–2.36)	0.000
No	24.3	1.00	
Asymptomatic Yes	7.6	1.00	
No	34.9	4.60 (2.05–10.3)	0.000
Medical consultation Yes	51.1	2.61 (1.95–3.50)	0.000
No	19.5	1.00	

^1^ Adjusted for age, sex, ABO blood groups, COVID-19 exposures, lifestyle, obesity (body mass index ≥ 30), occupation, chronic disease.

**Table 4:** Symptoms of acute COVID-19 illness and health recovery by adjusted incidence rate (aIR) and adjusted relative risk (aRR). Borriana COVID-19 cohort 2020.

Symptoms	Health Recovery		Total (%)
	aIR ^1^ (%)	aRR ^1^ (95% CI)	*p*-Value
Cough Yes	79.1	1.00	
No	83.5	1.06 (0.97–1.15)	0.237
Runny nose Yes	80.6	1.00	
No	83.4	1.04 (0.94–1.14)	0.481
Throat pain Yes	85.3	1.00	
No	79.9	0.94 (0.86–1.04)	0.148
Fever Yes	79.2	1.00	
No	84.8	1.07 (0.98–1.17)	0.123
Loss of smell/taste Yes	76.8	1.00	
No	86.9	1.13 (1.03–1.24)	0.007
Diarrhea Yes	77.8	1.00	
No	83.8	1.08 (0.96–1.20)	0.184
Vomits Yes	69.2	1.00	
No	82.7	1.20 (0.95–1.50)	0.127
Weakness Yes	78.7	1.00	
No	88.1	1.12 (1.02–1.22)	0.012
Headache Yes	77.7	1.00	
No	85.7	1.10 (1.01–1.21)	0.035
Myalgia Yes	80.2	1.00	
No	82.6	1.03 (0.94–1.13)	0.539
Dyspnea Yes	41.8	1.00	
No	75.8	1.81 (1.10–2.99)	0.020
Skin’s lesions Yes	76.2	1.00	
No	82.5	1.08 (0.94–1.25)	0.265
Number of symptoms ≥ 5 Yes	75.6	1.00	
No	86.8	1.15 (1.04–1.27)	0.006
Asymptomatic Yes	82.9	1.00	
No	81.0	0.98 (0.94–1.11)	0.576
Medical consultation Yes	69.9	1.00	
No	89.6	1.28 (1.15–1.42)	0.000

^1^ Adjusted for age, sex, ABO blood groups, COVID-19 exposures, lifestyle, obesity (body mass index ≥ 30), occupation, and chronic disease.

**Table 5:** Symptoms of acute COVID-19 illness and return to the usual health by adjusted incidence ratio (aIR) and adjusted relative risk (aRR). Borriana COVID-19 cohort 2020.

Symptoms	Return to the Usual Health		Total (%)
	aIR ^1^ (%)	aRR ^1^ (95% CI)	*p*-Value
Cough Yes	81.7	1.00	
No	83.9	1.03 (0.94–1.12)	0.528
Runny nose Yes	82.0	1.00	
No	84.5	1.03 (0.94–1.13)	0.499
Throat pain Yes	82.2	1.00	
No	85.3	1.03 (0.89–1.05)	0.438
Fever Yes	79.8	1.00	
No	85.3	1.07 (0.98–1.16)	0.142
Loss smell/taste Yes	79.5	1.00	
No	87.1	1.10 (1.01–1.19)	0.031
Diarrhea Yes	79.4	1.00	
No	84.8	1.07 (0.97–1.18)	0.196
Vomits Yes	86.3	1.00	
No	83.4	1.03 (0.88–1.06)	0.487
Weakness Yes	78.3	1.00	
No	90.2	1.15 (1.06–1.26)	0.001
Headache Yes	79.0	1.00	
No	87.1	1.10 (1.01–1.20)	0.025
Myalgia Yes	79.0	1.00	
No	85.2	1.07 (0.98–1.19)	0.112
Dyspnea Yes	73.4	1.00	
No	79.3	1.08 (0.68–1.71)	0.794
Skin’s lesions Yes	72.3	1.00	
No	84.4	1.17 (0.99–1.37)	0.061
Number of symptoms ≥ 5 Yes	77.2	1.00	
No	87.1	1.13 (1.03–1.24)	0.013
Asymptomatic Yes	87.7	1.00	
No	82.2	1.08 (0.97–1.19)	0.133
Medical consultation Yes	73.9	1.00	
No	90.9	1.22 (1.12–1.35)	0.000

^1^ Adjusted for age, sex, ABO blood groups, COVID-19 exposures, lifestyle, obesity (body mass index ≥ 30), occupation, chronic disease.

**Table 6:** Complications, risk factors, adjusted incidence ratio (aIR) and adjusted relative risk (aRR). Borriana COVID-19 cohort 2020.

Variables	Complications		
	aIR (%)	aRR (95% CI ^1^)	*p* Value
Sex ^2^ Female	40.6	1.93 (1.41–2.65)	0.000
Male	21.0	1.00	
Age-groups (years) ^3^			
0–34	25.8	1.00	0.004
35 and above	38.7	1.50 (1.14–1.99)	
ABO blood group ^4^			
O	31.2	1.00	-
A	32.2	1.03 (0.79–1.36)	0.816
B	46.9	1.51 (1.04–2.16)	0.026
AB	23.6	0.76 (0.31–1.86)	0.545
Occupations I–II ^5^	33.8	1.02 (0.78–1.33)	
III–VI	33.0	1.00	0.859
Chronic disease ^6^ Yes	35.8	1.12 (0.85–1.49)	0.416
No	31.9	1.00	
No-smoking ^7^	33.8	1.33 (0.88–2.02)	0.173
Ex-smoking	25.3	1.00	
Current smoking	40.7	1.61 (1.02–2.54)	0.041
Alcohol consumption ^8^ Yes	32.4	0.96 (0.70–1.32)	0.818
No	33.6	1.00	
Physical exercise ^9^ Yes	34.8	1.06 (0.73–1.22)	0.650
No	32.8	1.00	
Body Mass Index (BMI) (kg/m^2^) ^10^			
BMI ≥ 30.0 (Obesity)	36.7	1.12 (0.74–1.48)	0.781
BMI < 30	34.9	1.00	
COVID-19 exposure			
Observe a person with a cough at MGEs ^11,12^ Yes	41.7	1.38 (1.05–1.81)	0.022
No	30.2	1.00	
Contact with COVID-19 patient ^13^ Yes	34.2	1.06 (0.71–1.60)	0.767
No	32.3	1.00	
Family with COVID-19 patient ^14^ Yes	35.5	1.19 (0.91–1.55)	0.204
No	29.9	1.00	
Attendance MGEs ≥ 2 ^15^ Yes	36.4	1.42 (1.04–1.94)	0.003
No	25.6	1.00	
At least a COVID-19 exposure ^16^ Yes	34.3	2.13 (1.11–4.09)	0.023
No	16.1		

^1^ CI, confidence interval; ^2^ Adjusted for age ABO blood group; ^3^ Adjusted for sex ABO blood group; ^4^ Adjusted for age sex; ^5^ Adjusted for age sex ABO blood goup; ^6^ Adjusted for age sex ABO blood group occupation lifestyle obesity; ^7^ Adjusted for age sex ABO blood group occupation lifestyle obesity; ^8^ Adjusted for age sex ABO blood group occupation lifestyle obesity; ^9^ Adjusted for age sex ABO blood group occupation lifestyle obesity information 14; ^10^ Adjusted age sex ABO blood group occupation lifestyle; ^11^ MGEs, mass gathering events; ^12^ Adjusted for age sex ABO blood group chronic disease occupation obesity other COVID-19 exposures; ^13^ Adjusted for age sex ABO blood group chronic disease occupation obesity other COVID-19 exposures; ^14^ Adjusted for age sex ABO blood group chronic disease occupation obesity other COVID-19 exposures; ^15^ Adjusted for age sex ABO blood group chronic disease occupation obesity other COVID-19 exposures; ^16^ Adjusted for age sex ABO blood group chronic disease occupation obesity.

**Table 7:** Health recovery, risk factors, adjusted incidence ratio (aIR) and adjusted relative risk (aRR).Borriana COVID-19 cohort 2020.

Variables	Health Recovery		
	aIR (%)	aRR (95% CI ^1^)	*p*-Value
Sex ^2^ Female	78.7	1.00	
Male	85.8	1.09 (1.00–1.19)	0.046
Age-groups (years) ^3^			
0–34	87.7	1.15 (1.06–1.25)	0.001
35 and above	76.3	1.00	
ABO blood group ^4^			
O	83.0	1.00	
A	81.6	1.12 (0.94–1.33)	0.211
B	74.3	1.10 (0.92–1.31)	0.301
AB	100.0	1.35 (1.14–1.58)	0.000
Occupations I–II ^5^	84.6	1.06 (0.97–1.15)	0.245
III–VI	80.4	1.00	
Chronic disease ^6^ Yes	80.1	1.00	
No	84.0	1.05 (0.96–1.15)	0.286
No-smoking ^7^	83.4	1.02 (0.91–1.15)	0.686
Ex-smoking	81.5	1.00	
Current smoking	78.4	1.04 (0.82–1.13)	0.634
Alcohol consumption ^8^ Yes	78.7	1.00	
No	82.5	1.04 (0.94–1.17)	0.406
Physical exercise ^9^ Yes	80.2	1.00	
No	82.2	1.03 (0.94–1.12)	0.518
Body Mass Index (BMI) (kg/m^2^) ^10^			
BMI ≥ 30.0 (Obesity)	79.8	1.00	
BMI < 30	83.9	1.05 (0.96–1.15)	0.270
Observe a person with a cough at MGEs ^11,12^ Yes	79.0	1.00	
No	83.1	1.05 (0.94–1.16)	0.374
Contact with COVID-19 patient ^13^ Yes	81.2	1.00	
No	88.4	1.09 (0.98–1.21)	0.124
Family with COVID-19 patient ^14^ Yes	80.7	1.00	
No	85.4	1.06 (0.97–1.15)	0.181
Attendance MGEs ≥ 2 ^15^ Yes	78.9	1.00	
No	87.8	1.11 (1.02–1.21)	0.014
At least a COVID-19 exposure ^16^ Yes	81.2	1.00	
No	82.1	1.01 (0.85–1.20)	0.904

^1^ CI, confidence interval; ^2^ Adjusted for age ABO blood group; ^3^ Adjusted for sex ABO blood group; ^4^ Adjusted for age sex; ^5^ Adjusted for age sex ABO blood goup; ^6^ Adjusted for age sex ABO blood group occupation lifestyle obesity; ^7^ Adjusted for age sex ABO blood group occupation lifestyle obesity; ^8^ Adjusted for age sex ABO blood group occupation lifestyle obesity; ^9^ Adjusted for age sex ABO blood group occupation lifestyle obesity information 14; ^10^ Adjusted age sex ABO blood group occupation lifestyle; ^11^ MGEs, mass gathering events; ^12^ Adjusted for age sex ABO blood group chronic disease occupation obesity other COVID-19 exposures; ^13^ Adjusted for age sex ABO blood group chronic disease occupation lifestyle obesity other COVID-19 exposures; ^14^ Adjusted for age sex ABO blood group chronic disease lifestyle occupation obesity other COVID-19 exposures; ^15^ Adjusted for age sex ABO blood group chronic disease lifestyle occupation obesity other COVID-19 exposures; ^16^ Adjusted for age sex ABO blood group chronic disease lifestyle occupation obesity.

**Table 8:** Return to the usual health, risk factors, adjusted incidence ratio (aIR) and adjusted relative risk (aRR). Borriana COVID-19 cohort 2020.

Variables	Return to the Usual Health		
	aIR (%)	aRR (95% CI ^1^)	*p*-Value
Sex ^2^ Female	79.5	1.00	
Male	87.9	1.11 (1.02–1.20)	0.014
Age-groups (years) ^3^			
0–34	88.9	1.14 (1.05–1.24)	0.002
35 and above	78.0	1.00	
ABO blood group ^4^			
O	86.7	1.00	
A	81.0	0.93 (0.86–1.01)	0.094
B	75.7	0.87 (0.73–1.04)	0.128
AB	100.0	1.15 (1.09–1.21)	0.000
Occupations I–II ^5^	86.2	1.05 (0.97–1.14)	0.219
III–VI	81.9	1.00	
Chronic disease ^6^ Yes	82.3	1.00	
No	85.0	1.03 (0.95–1.12)	0.449
No-smoking ^7^	84.0	1.03 (0.92–1.16)	0.565
Ex-smoking	81.0	1.00	
Current smoking	87.0	1.08 (0.94–1.23)	0.266
Alcohol consumption ^8^ Yes	82.6	1.00	0.737
No	84.0	1.02 (0.92–1.12)	
Physical exercise ^9^ Yes	82.0	1.00	
No	82.6	1.00 (0.92–1.08)	0.920
Body Mass Index (BMI) (kg/m^2^) ^10^			
BMI ≥ 30.0 (Obesity)	80.0	1.00	0.659
BMI < 30	82.4	1.03 (0.85–1.11)	
Observe a person with a cough at MGEs ^11,12^ Yes	78.9	1.00	
No	85.1	1.08 (0.97–1.19)	0.146
Contact with COVID-19 patient ^13^ Yes	81.6	1.00	
No	94.0	1.15 (1.06–1.25)	0.000
Family with COVID-19 patient ^14^ Yes	81.8	1.00	
No	84.8	1.04 (0.95–1.13)	0.389
Attendance MGEs ≥ 2 ^15^ Yes	82.0	1.00	
No	86.7	1.07 (0.97–1.15)	0.194
At least a COVID-19 exposure ^16^ Yes	82.4	1.00	
No	91.1	1.11 (0.99–1.22)	0.056

^1^ CI, confidence interval; ^2^ Adjusted for age ABO blood group; ^3^ Adjusted for sex ABO blood group; ^4^ Adjusted for age sex; ^5^ Adjusted for age sex ABO blood goup; ^6^ Adjusted for age sex ABO blood group occupation lifestyle obesity; ^7^ Adjusted for age sex ABO blood group occupation lifestyle obesity; ^8^ Adjusted for age sex ABO blood group occupation lifestyle obesity; ^9^ Adjusted for age sex ABO blood group occupation lifestyle obesity information 14; ^10^ Adjusted age sex ABO blood group occupation lifestyle; ^11^ MGEs, mass gathering events; ^12^ Adjusted for age sex ABO blood group chronic disease occupation obesity other COVID-19 exposures; ^13^ Adjusted for age sex ABO blood group chronic disease occupation obesity other COVID-19 exposures; ^14^ Adjusted for age sex ABO blood group chronic disease occupation obesity other COVID-19 exposures; ^15^ Adjusted for age sex ABO blood group chronic disease occupation obesity other COVID-19 exposures; ^16^ Adjusted for age sex ABO blood group chronic disease occupation obesity.

## Data Availability

Data of the study can be consulted if the authors are requested. Dataset: borrianacohort.dta.
